# Epigenetic Programming and Fetal Metabolic Programming

**DOI:** 10.3389/fendo.2019.00764

**Published:** 2019-12-03

**Authors:** Ziqiang Zhu, Fang Cao, Xiaozhong Li

**Affiliations:** ^1^Children's Hospital of Soochow University, Suzhou, China; ^2^Changzhou Maternity and Child Health Care Hospital affiliated to Nanjing Medical University, Changzhou, China

**Keywords:** epigenetic programming, fetal metabolic programming, metabolic syndrome, obesity, insulin resistance, adverse intrauterine environment

## Abstract

Fetal metabolic programming caused by the adverse intrauterine environment can induce metabolic syndrome in adult offspring. Adverse intrauterine environment introduces fetal long-term relatively irreversible changes in organs and metabolism, and thus causes fetal metabolic programming leading metabolic syndrome in adult offspring. Fetal metabolic programming of obesity and insulin resistance plays a key role in this process. The mechanism of fetal metabolic programming is still not very clear. It is suggested that epigenetic programming, also induced by the adverse intrauterine environment, is a critical underlying mechanism of fetal metabolic programming. Fetal epigenetic programming affects gene expression changes and cellular function through epigenetic modifications without DNA nucleotide sequence changes. Epigenetic modifications can be relatively stably retained and transmitted through mitosis and generations, and thereby induce the development of metabolic syndrome in adult offspring. This manuscript provides an overview of the critical role of epigenetic programming in fetal metabolic programming.

Metabolic syndrome is well-known as a syndrome involved in obesity, insulin resistance, impaired glucose tolerance/diabetes, disturbance of lipid metabolism, and cardiovascular complications. Metabolic syndrome includes at least three typical phenotypes as follow: (1) elevated abdominal obesity, (2) elevated fasting glucose, (3) elevated blood pressure, (4) elevated fasting triglycerides (TG), (5) reduced high-density lipoprotein cholesterol (HDL-C) (1). It is one of the most causes of morbidity and mortality in both the developed and developing countries.

Obesity and insulin resistance are key factors resulting metabolic syndrome (Figure 1). Prevalence of metabolic syndrome was thought to be mainly contributed by genetics under an unhealthy lifestyle in adult. However, genetics cannot explain all reasons causing metabolic syndrome. Nowadays, it is proposed that an adverse intrauterine environment can induce fetal metabolic programming of obesity and insulin resistance and further metabolic syndrome in adult offspring. Epigenetic programming, without DNA nucleotide sequence changes, is a critical underlying mechanism of fetal metabolic programming of metabolic syndrome in adult offspring (2–6). Intergenerational transmission of metabolic syndrome moves in cycles through fetal metabolic programming and fetal epigenetic programming as shown in Figure 2.

**Figure 1:**
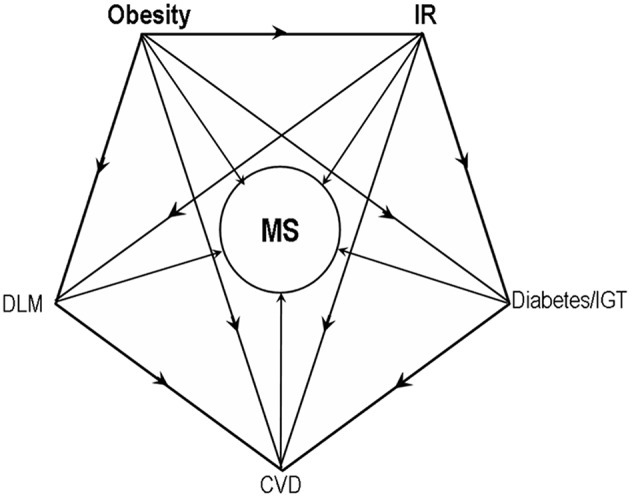
Obesity and insulin resistance as key factors resulting metabolic syndrome. IR, insulin resistance; IGT, Impaired glucose tolerance; DLM, disturbance of lipid metabolism; CVD, cardiovascular diseases.

**Figure 2:**
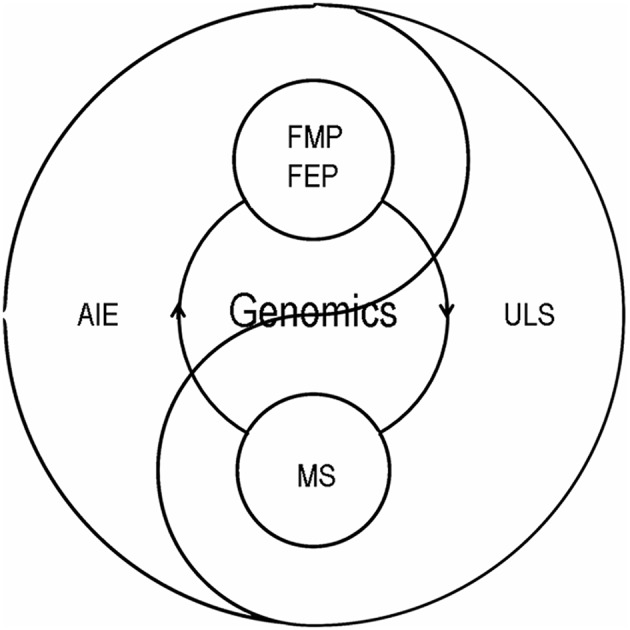
Intergenerational transmission of metabolic syndrome. Adverse intrauterine environment induced fetal epigenetic programming and fetal metabolic programming may introduce a circle of metabolic syndrome across generations without genetic changes. MS, metabolic syndrome; FMP, fetal metabolic programming; FEP, fetal epigenetic programming; AIE, adverse intrauterine environment; ULS, unhealthy lifestyle.

## Fetal Metabolic Programming

The concept of fetal programming was firstly proposed by Barker and Hales in 1998, and later became the theory of developmental origins of health and disease (DOHaD) (7). This theory proposes that fetal metabolic programming caused by adverse intrauterine environment induces offspring metabolic diseases in adults. Fetal metabolic programming is involved in the relatively permanent changes of metabolic signaling pathways in growth and development, leading to relatively nonreversible effects on the structure and/or functional changes of fetal growth and development (8–10).

Although fetal metabolic programming is a relatively new concept, there have been various supporting evidence. It is proposed that the adverse intrauterine environment can cause intrauterine growth retardation (IGR), small for gestational age (SGA) and further metabolic syndrome risk in adult offspring. The intrauterine environment of malnutrition or nutrition deficiency can induce such fetal abnormal growth which is associated with metabolic syndrome in adult offspring (11). On the other hand, similar to intrauterine malnutrition, intrauterine over-nutrition exposure, such as in obesity or gestational diabetes, can also cause adverse fetal metabolic programming, leading to neonatal SGA or Large for gestational age (LGA) and further metabolic syndrome in adults (12–14).

Adverse intrauterine environment could introduce fetal long-term relatively irreversible changes in organs and metabolism, and thus may cause fetal metabolic programming of obesity and insulin resistance, leading metabolic syndrome in adult offspring.

### Fetal Programming of Obesity

#### Human Studies

Offspring obesity in adults can be caused by intrauterine malnutrition exposure. This concept was first proposed in a study that the male offspring's obesity rate in adult was significantly associated with severe malnutrition in pregnant women in 40s Holland famine in the last century (15). Similar findings were observed in other countries (16). Also, adult obesity risk of low birth weight neonates was higher than normal weight neonates.

On the other hand, maternal over-nutrition or obesity is also highly correlated with offspring obesity (17–21). It is also proposed that pregnancy weight gain, independent of maternal obesity, is an independent factor causing neonatal adipose increase and subsequent obesity in children and adults (22). Maternal insulin resistance and glucose levels were reported to be important mediators of maternal obesity associated neonatal adipose increase (23). Otherwise, hyperglycemia in gestational diabetes could induce the risk of SGA or LGA.

#### Animal Model Studies

In the animal model studies, factors causing the prevalence of obesity in offspring can be elaborately studied by gestational interventions such as diet, drugs, surgery, and others. Animal model studies also offer an opportunity to disclose the detailed mechanism of fetal development programming.

In agreement with human studies, it was reported that offspring obesity could be induced by maternal malnutrition or over-nutrition during pregnancy, in studies in mice, rats, sheep, and non-human primates. Offspring obesity has been reported to be related to the significant expression changes of genes involved in abnormal metabolism, such as mitochondrial dysfunction (24) and significantly increased stress response (25, 26).

Rodent animal experiments have reported that fetal developmental programming caused by adverse intrauterine environment could induce hyperphagia leading offspring obesity in adults. Additional studies reported that the factors causing this offspring hyperphagia included hypothalamic programming of leptin resistance, increased neuropeptide Y and decreased proopiomelanocortin (27–30). It was reported that fetal development programming also introduced changes in offspring behavior patterns (sedentary and reduced physical activity), which contributed to the development of obesity in adults (31, 32).

### Fetal Programming of Hyperinsulinemia and Insulin Resistance

#### Human Studies

The concept of fetal/neonatal hyperinsulinemia in pregnant women with gestational diabetes mellitus has been proposed a long time ago (33). Intrauterine hyperglycemia in gestational diabetes mellitus can induce fetal/neonatal hyperinsulinemia. Fetal/Neonatal hyperinsulinemia help to induce LGA/macrosomia. Also, fetal/neonatal hyperinsulinemia induced neonatal hypoglycemia in earlier hours after birth.

It is suggested that neonatal hyperinsulinemia may not only be a transient phenomenon caused by neonatal hyperglycemia but also reflect fetal or neonatal insulin resistance which would induce metabolic syndrome in adults. For one thing, fetal/neonatal hyperinsulinemia can be introduced by maternal obesity, higher weight gain, a high-fat diet or other factors (34–36). For another thing, it was reported that fetal/neonatal hyperinsulinemia could also be induced in glucose-well-controlled pregnant women with gestational diabetes (37, 38).

Fetal or neonatal insulin resistance was newly reported to be induced by gestational diabetes (37–41). Otherwise, fetal/neonatal insulin resistance can also be induced without intrauterine hyperglycemia exposure. For example, insulin resistance without hyperglycemia was reported in preterm neonates (42). Fetal insulin resistance was associated with maternal insulin resistance or insulin sensitivity in non-diabetic pregnancies (34, 43). Fetal/neonatal hyperinsulinemia could be induced in glucose-well-controlled pregnant women with gestational diabetes (37, 38). Fetal/neonatal insulin resistance was reported to be induced by maternal obesity (35, 44). Also, LGA was reported to be associated with decreased fetal Insulin sensitivity (39). Programming of fetal insulin resistance was reported to be induced by intrauterine abnormal activation of inflammation, adipokines and the endoplasmic reticulum (ER) stress (35).

#### Animal Model Studies

It has been reported by animal experiments that various maternal interventions could introduce hyperinsulinemia or insulin resistance in offspring.

Earlier studies proposed that fetal/neonatal hyperinsulinemia might be directly caused by fetal/neonatal simultaneous hyperglycemia. However, it was reported in some animal experiments that hyperinsulinemia can also be introduced without hyperglycemia in offspring. For example, fetal/neonatal hyperinsulinemia was reported to be induced by maternal hyperinsulinemia (45).

Further related animal experiments suggested that not only fetal/neonatal hyperinsulinemia but also fetal/neonatal insulin resistance can be induced by various maternal gestational interventions. It was reported that insulin resistance in offspring can be induced by gestational interventions such as high-fat diets (46–48), high-fructose diets (49), malnutrition or nutrition deficiency (50, 51), chronic intermittent hypoxia exposure (52, 53), abnormal levels of hormones (54–56), chemical or drug induction (57–59), and diabetic induction by genetic or non-genetic factors (60). Besides diabetes models, offspring hyperinsulinemia exists without maternal hyperglycemia in some other rodent animal experiments above (61, 62). Taken together, besides hyperglycemia, it is suggested that there is some other important potential mechanism causing fetal programming of insulin resistance.

Some adverse intrauterine environment also induces decreased number and/or function of pancreatic beta cells leading decreased glucose-stimulated insulin secretion and further abnormal glucose tolerance in adult offspring. For example, in animal experiments, the decreased number of pancreatic beta cells, introduced by maternal low-protein diet exposure, is caused by reduced activity of the mTOR signaling pathway (63). Besides, maternal low-protein diet exposure also introduces a decreased Pdx1 and Glut2 levels in offspring (64, 65). Both of them thereby may induce impaired glucose tolerance of offspring in adults.

It was recently reported that there was transgenerational programming of hyperinsulinemia, insulin resistance or glucose tolerance in various studies (4, 66, 67). For example, in several rodent studies, it has been reported that maternal high-fat diet induced obesity, insulin resistance, and glucose tolerance, not only in the first generation (F1) offspring but also in the second generation (F2) offspring (68, 69). In one of these studies above, it was observed that there were hyperinsulinemia and hyperleptinemia in the F2 generation, with normal body weight and glucose level (70). In another mouse model, it was reported that maternal obesogenic diet exposure induced hyperinsulinemia and expression alternation of some hepatic genes in the F2 offspring, interestingly without apparent phenotype in the F1 generation (71). Also, some fetal experiments in sheep reported similar results of transgenerational hyperinsulinemia, insulin resistance or glucose tolerance (72).

## Fetal Epigenetic Programming

The critical mechanism underlying fetal metabolic programming induced by adverse intrauterine environment is still not very clear, while fetal metabolic programming has been reported to be contributed by changes in the developmental trajectories of tissues, stem cell numbers, neural circuits, and so on. Epigenetic programming, also induced by the adverse intrauterine environment, was reported to play an important role in the differentiation and development of embryonic, tissue, and stem cells (73–76). Could it be a critical underlying mechanism of fetal metabolic programming?

Epigenetics is the inheritance of variation in gene expression without changes in the DNA nucleotide sequence. Epigenetic modification can be relatively stably transmitted in the process of cell proliferation. The known mechanisms include DNA methylation, histone modification, genomic imprinting, chromatin remodeling, non-coding RNA, and so on. Such mechanism regulates gene expression at levels of pre-transcription (heterochromatinization), transcription (chromatin activation, binding of the cis-acting element, and the trans-acting factor) and post-transcription (protein translation).

DNA methylation and histone modification are known as key epigenetic regulation factors underlying the regulation of chromatin structure and gene transcription. DNA methylation adds methyl on 5-cytosine by DNA methyltransferase (Dnmt) at cis-acting element including promoter, enhancer, regulatory regions and sequence, response element, and so on. DNA methylation on such regions can cause DNA conformational changes, which may affect the binding of trans-acting factor and thereby regulate genes expression. Histone modification is known as the “histone code” including histone methylation, acetylation, phosphorylation, adenylation, ubiquitination, and so on. Histone modifications affect the structure and function of chromatin and thereby regulate the expression of the located genes.

Genomic imprinting, a parent-of-origin-specific silencing manner of certain gene alleles, is established (“imprinted”) in the germline (sperm or egg cells) mainly through marks of DNA methylation and histone modification. Non-coding RNA can be divided into long chain non-coding RNA (lncRNA) and short chain non-coding RNA (sncRNA), including miRNA, siRNA, piRNA, dsRNA, Xist RNA, Tsix RNA, telomere RNA, and so on. Micro RNA (miRNA) is the main executor of RNA interference effect on gene expression. LncRNA plays an important role in cell differentiation and individual development by multiple methods. It is involved in X chromosome silencing, genomic imprinting, chromatin modification, activation or inhibition of transcription, transportation in nuclear, and other unknown functions.

The concept of epigenetic programming was proposed as the potential key role of epigenetic modification in fetal metabolic programming (77, 78). Epigenetic modification without genetic changes is more easily induced by the adverse intrauterine environment during germ cells and fetal development, and relatively stably transmitted until adults (79). Epigenetic modification induced changes in cellular or tissue function can thereby be relatively stably transmitted earlier from fetuses until adults, and induce the development of metabolic syndrome in adult offspring. There is a sensitive “window” for epigenetic programming when germ cells, embryos, and fetuses are developing.

### Epigenetic Programming of Obesity

#### Human Studies

Epigenetic programming of obesity was reported in human studies to be involved in abnormal fetal growth, adipocytokines, and other obesity-associated genes.

Global genome-wide epigenetic changes in offspring were reported to be associated with offspring obesity or an adverse intrauterine environment. Firstly, gestational diabetes induced genome-wide methylation changes in neonatal cord blood (80–82), adipose tissue or placenta (83). Genome-wide methylation changes were also reported existing in peripheral blood leukocytes of offspring at children (84, 85) or adult (86) exposed to the diabetic intrauterine environment. In addition, changes of global DNA methylation signatures were reported to exist in adult offspring under prenatal famine exposure in Dutch Hunger Winter and link to growth and metabolism (87). DNA methylation of metastable epialleles in neonatal lymphocytes and hair follicles was reported to be modulated by maternal nutrition at conception induced by Gambian Seasonal diet (88). Secondly, it was reported that maternal obesity was associated with genome-wide epigenetic changes in children or adult offspring (84, 89). Thirdly, DNA methylation changes in the placenta or the cord Blood were reported to be associated with abnormal birthweight or BMI in childhood (90, 91). Birth weight was also reported to be associated with methylation changes in children and adult offspring (92). What's more, circulating extracellular RNA ex- miR-122 was reported to be associated with regional adiposity in adults (93). Lastly, other adverse intrauterine environments, such as Arsenic or tobacco exposure, were also reported to be associated with genome-wide DNA methylation in cord blood (94, 95).

The insulin-like growth factor 2 (IGF2) and H19 genes are imprinted genes. Hypomethylation of the IGF2 gene and hypermethylation of H19 gene are associated with significantly higher expression of IGF2 (lower transcribed of H19), which induce neonatal adiposity through growth promoting effects. Such methylation changes of IGF2 and H19 in umbilical cord blood and placenta were reported to be associated with macrosomia exposed to GDM induced intrauterine hyperglycemia (96). Lower DNA methylation of the imprinted IGF2 gene was also reported in individuals who were prenatally exposed to famine during the Dutch Hunger Winter compared with their unexposed, same-sex siblings, supporting for the hypothesis that epigenetic changes induced by adverse intrauterine environment could persist throughout life (97). Changes in DNA methylation of the IGF2 gene was reported in cord blood from female offspring of pregnant women with an intervention of folic acid supplementation (98) or in blood from neonates of physically active pregnant women (99) than the control groups. In addition, in adult monozygotic twins with discordant birthweight, a significant positive association existed between birth weight and IGF1R DNA methylation differences in adult blood. It was suggested that intra-uterine growth differences were associated with methylation changes in the IGF1R gene in adulthood, independent of genetic effects (100). Higher methylation and lower expression of proopiomelanocortin (POMC) in cord blood was associated with lower birth weight and higher triglycerides in children blood, suggesting an early predictive marker of future metabolic syndrome (101).

Leptin could increase adipose tissue lipolysisl and hepatic β-oxidation of fatty acids, and inhibit hyperphagia. Hypermethylation and lower expression of the letpin gene in placenta and cord blood were reported to be associated with maternal impaired glucose tolerance (IGT) (102), GDM (103), or obesity (104). On the contrary, Lower DNA methylation and higher expression of the leptin gene were reported in the blood of obese children (105). Such inconsistency may be contributed by the differences between the intrauterine environment and the environment after birth. More interesting, the higher level of leptin cannot inhibit hyperphagia because of the leptin resistance. Adiponectin could increase GLUT4- mediated glucose uptake in adipocyte, resulting in adipogenesis and adipocyte lipid storage. Hypermethylation of adiponectin gene in newborn blood was reported in GDM group (106).

It was reported that abnormal birth weight was associated with cord blood or placental DNA methylation changes in energy homeostasis genes (107) and LINE-1 (91). It was reported that hypermethylations and lower expressions of the ATG2B, NKX6.1, and SLC13A5 genes (respectively, related to autophagy, beta-cell development and function, and lipid metabolism) and hypomethylation and higher expression of GPR120 gene (related to free fatty acid regulation) existed in the placenta and cord blood from SGA newborns (107). Abnormal birth weight was also reported to be related to placental methylation changes in WNT2 gene (108), Glucocorticoid receptor gene (109), fat mass- and obesity-associated genes (110), and Cardiometabolic Risk genes (111). In gestational diabetes, it was reported that hypermethylations and lower expressions of the lipoprotein lipase gene in the placenta are associated with offspring body composition at 5 years of age (112).

#### Studies in Animal Models or *in vitro* Models

Animal models and *in vitro* models disclosed more details of epigenetic programming of obesity in various tissues, which was involved in DNA or histone-modifying enzyme, abnormal fetal growth, hyperphagia, energy balance regulation, adipocyte differentiation/maturity, adipocytokines, hepatic metabolism, microRNA, and so on.

In liver, muscle and adipose tissues of offspring mice, global DNA methylation was reported to be induced by a maternal high-fat diet (113). A maternal high-fat diet induced epigenetically alters in fetal hepatic chromatin structure in primates by histone modifications and hence lends a molecular basis to the fetal origins of adult disease hypothesis (114). Non-human primate fetal hepatic multiple pathway dysregulation was reported to be associated with marked lipid accumulation, in response to maternal obesity induced by a high-fat, high-fructose diet prior to pregnancy, by unbiased gene and microRNA abundance analyses (115). DNA methylation levels in human *in vitro* maturation (IVM) oocytes were reported to be changed by High-glucose concentrations (116).

The fetal hepatic and placental expression of epigenetic machinery genes, particularly histone acetylation pathway genes, was reported to be sensitive to high-fat-diet-induced maternal obesity, leading to fetal growth restriction (FGR) in mice (117). More interesting, maternal weight loss appears beneficial to fetal growth, but those maternal obesity-induced effects were retained in offspring. Maternal high-fat diet increases fetal hepatic H3K14 acetylation with concomitant decreased SIRT1 expression and *in vitro* deacetylase activity in non-human primates (118). The protein deacetylase sirtuin-1 (SIRT1) was reported to be thought as a potential central co-ordinator of nutrient-led short and longer-term programming of tissue function (119).

Similar to human studies, the methylation changes in the imprinted gene IGF2/H19 was reported being an important factor involved in abnormal birth weight in various animal models (120–122). Decreased Histone modifications and higher expression of hepatic gene IGF1 were also reported in IUGR rat (123). In this study, the modulation of the rate of IUGR newborn catch-up growth may thus protect against IGF1 epigenetic modifications and, consequently, obesity and associated metabolic abnormalities.

Epigenetic programming of hyperphagia in offspring plays a role in the fetal programming of obesity. Leptin inhibits hyperphagia and stimulates energy expenditure through interactions with neuronal pathways in the hypothalamus. Leptin is higher in obese subjects. It was also reported that higher expression of the leptin gene, associated with higher histone or DNA methylation, was reported to be induced by maternal high-fat diet exposure (124) and to be involved in the transgenerational obesity (125). However, such obesogenic hyperphagia above is not suppressed because leptin resistance could be induced by epigenetic regulation of the leptin signaling circuit (126).

Epigenetic changes in the mouse adiponectin gene promoters were also reported to be associated with offspring obesity induced by paternal high-fat diet exposure (127). Lower adiponectin level and lower histone acetylation and higher histone methylation levels of the adiponectin gene promoter were reported in adipose tissues of mouse offspring with a high-fat-diet exposure during pregnancy (124).

Maternal periconceptional undernutrition induced decreased DNA methylation and increased histone acetylation was reported in pro-opiomelanocortin (POMC) (128) and the glucocorticoid receptor (GR) (129) in ovine fetal hypothalamus. Such epigenetic changes were associated with reduced POMC and increased GR levels, which potentially resulted in the altered energy balance regulation in the offspring. Another study reported that the hypermethylation and decreased expression of pro-opiomelanocortin gene caused hyperphagic obesity in mouse offspring under pregnant triclosan exposure (130). In the female hypothalamic paraventricular nucleus, it was reported that a perinatal high-fat diet environment induced decreased melanocortin 4 receptor (Mc4r) which was associated with food intake and energy balance (131). Such Mc4r downregulation may be contributed by histone acetylation of its promoter binding thyroid hormone receptor-β (TRβ), an inhibitor of Mc4r transcription, by using chromatin immunoprecipitation and bisulfite sequencing.

Epigenetic programming plays a role in adipocyte differentiation and maturity leading offspring obesity. DNA (cytosine-5) methyltransferase 3a (Dnmt3a) in 3T3-L1 preadipocytes was reported to be transcriptionally upregulated by Activator protein 2alpha (AP2alpha) through directly binding to its proximal promoter region, leading to increased promoter methylation of adipogenic genes which were required for granting preadipocyte the ability to differentiate (132). Also, it was reported that DNA methylation of obesity-related genes in adipose-derived stem cells in low birthweight may programme the mature adipocyte function which influences the risk of metabolic diseases (133). Decreased beige adipocyte number and mitochondrial respiration were reported to coincide with increased histone methyltransferase (G9a) and reduced FGF21 gene expression in subcutaneous adipose tissue of rats fed prenatal low protein and postnatal high-fat diets (134). Significantly enhanced hepatic acetylation of histone H3 surrounding fatty acid synthase (FAS) gene promoter was reported in adult male offspring under fetal and neonatal exposure to nicotine, associated with increased expression of fatty acid synthase induced augmented hepatic and circulating triglycerides (135). DNA and histone methylation modifications of Zfp423 gene were reported to be induced by maternal obesity and facilitate Zfp423 expression and enhance adipogenic differentiation in fetal mice adipose tissue (136).

A high-fat diet during pregnancy was reported to induce neonatal gender-specific hepatic fat accumulation by increased Phosphoenolpyruvate carboxykinase (PEPCK) expression and histone modification (137). These alterations were reported to persistent in adult offspring by further studies from the same team (135, 138). Developmental bisphenol A (BPA) exposure sex-specifically altered DNA methylation and histone marks (H3Ac, H4Ac, H3Me2K4, H3Me3K36), and decreased the binding of several transcription factors (Pol II, C/EBPβ, SREBP1) within the male Cpt1a gene, the key β-oxidation enzyme, which may exacerbate high-fat diet-induced hepatic steatosis (139). It was also reported that gestational high-fat diet programs hepatic phosphoenolpyruvate carboxykinase gene expression and histone modification in neonatal offspring rats (137) and female adult offspring rats (138). Another study reported that histone demethylase Plant Homeodomain Finger 2 (Phf2) was a new transcriptional co-activator of the transcription factor Carbohydrate Responsive Element (ChRE) Binding Protein, and acted as a molecular checkpoint to prevent NAFLD progression during obesity (140).

Changes of microRNA was reported to be induced by the adverse intrauterine environment and associated with offspring obesity (141). For example, maternal obesity-induced miRNA-let-7g downregulation in fetal skeletal muscle was reported to may enhance intramuscular adipogenesis during ovine fetal muscle development (142).

### Epigenetic Programming of Insulin Resistance

#### Human Studies

Epigenetic programming of insulin resistance in human studies was reported to be involved in the epigenetic modifying enzyme, inflammatory/proinflammatory factors, insulin associated signaling, energy balance regulation, and microRNA.

Global genome-wide epigenetic changes were reported to be associated with insulin resistance. DNA methylation levels in neonatal blood were reported to be associated with insulin sensitivity in early childhood (90). In whole-blood of adults, potential DNA methylation biomarkers changes were reported to be strongly associated with obesity and insulin resistance (143). In visceral adipose tissue of obese subjects, it was reported that genome-wide DNA methylation pattern could differentiate insulin-resistant from insulin-sensitive (144). In the human liver of subjects with non-alcoholic steatohepatitis, methylation alterations are related to insulin resistance (145).

Changes of histone deacetylase 3 (HDAC3) in peripheral blood mononuclear cells is reported to be strongly related to insulin resistance and related proinflammatory mediators in patients with type 2 diabetes (143, 146).

Higher DNA methylation changes in neonatal adiponectin gene were reported in gestational diabetes group (103, 106). It was reported that IR was correlated negatively with DNA methylation of complement factor C3 gene, and correlated positively with the levels of complement factor C3, in adipose tissue from obese subjects (147). DNA methylation of the lymphocyte antigen 86 (LY86) gene was reported to be associated with obesity, and insulin resistance through a multi-stage cross-sectional study (148).

In white adipose tissue in insulin-resistant obese women, significantly differentially methylated sites were reported in IR-associated genes in pathways related to integrin cell surface interactions and insulin signaling pathway (149). In adult peripheral white blood cells, methylation changes of the endoplasmic reticulum (ER) genes were also reported to be associated with insulin resistance (150). DNA methylation of hypoxia-inducible factor 3-alpha (HIF3A) in both blood cells and subcutaneous adipose tissue (SAT) was reported to be associated with BMI and whole-body insulin sensitivity (151). Fatty acyl CoA reductase 2 (FAR2) gene methylation changes in peripheral blood cells were reported to be associated with atypical antipsychotic-induced insulin resistance and lipid metabolism (152). Higher pro-opiomelanocortin (POMC) methylation in cord blood was associated with hyperinsulinemia in children blood, suggesting an early predictive marker of future metabolic syndrome (101).

Expression of IR associated microRNA-15a and microRNA-15b was reported to be increased in skeletal muscle from adult offspring of women with diabetes in pregnancy (153). Circulating extracellular RNA ex-miR-122 was reported in another study to be associated with IR in children and adults (93).

#### Studies in Animal Models or *in vitro* Models

Epigenetic programming of insulin resistance was disclosed in more details involved in DNA or histone-modifying enzyme, adipocytokines, insulin associated signaling, lipid metabolism, microRNA, and so on.

Global changes of decreased hepatic DNA methylation and H4 acetylation or increased hepatic H3 acetylation was reported in offspring exposed to gestational germinated brown rice and its gamma-amino-butyric acid-rich, which could prevent high-fat-diet-induced insulin resistance in first generation rat offspring (154). Hepatic H3K14ac and H3K9me3 were reported to be significantly increased in WT and G4 (±) fetal and 5-week murine offspring with HFD exposure *in utero* (155).

Adipose DNA methyltransferase 3a (Dnmt3a) was reported to be a novel epigenetic mediator of insulin resistance *in vitro* and *in vivo* (156). In cultured mouse and human adipocytes, Dnmt3a was reported to be both necessary and sufficient to mediate diet-induced insulin resistance. Adipose-specific Dnmt3a knock-out mice were reported to be protected from diet-induced insulin resistance and glucose intolerance without accompanying changes in adiposity. Fgf21, a key negatively regulated Dnmt3a target gene, can rescue Dnmt3a-mediated insulin resistance. Consistent with this, DNA methylation at the FGF21 locus was elevated and negatively correlated with adipose FGF21 expression in human subjects with diabetes.

Epigenetic changes in the adiponectin gene play an important role in mediating insulin resistance. It was reported that insulin resistance was mediated by obesity-induced DNA hypermethylation of the adiponectin gene (157). It was reported in adipose tissue from HFD-induced obese mice that obesity-induced suppression of adiponectin expression was induced by the increased DNMT1 expression/enzymatic activity and related DNA methylation and chromatin remodeling in the adiponectin gene promoter, and could be stimulated by DNMT inhibitor with amelioration of obesity-induced glucose intolerance and insulin resistance in an adiponectin-dependent manner. However, little is known about how does DNMT1 selectively methylate adiponectin gene, which is a critical issue in fetal epigenetic programming. Another research reported that gestational sleep fragmentation induced insulin resistance and epigenetic changes in adiponectin gene in visceral white adipose tissue (VWAT) of male adult offspring mice (158). Reductions in 5-hydroxymethylcytosine and H3K4m3 and an increase in DNA 5-methylcytosine and H3K9m2 in the promoter and enhancer regions of adiponectin gene emerged in adipocytes from VWAT and correlated with adiponectin gene expression, accompanied with increased DNMT3a/b and reduced histone acetyltransferase activity and TET1/2/3 expression.

In liver and muscle tissues of rats, it was reported that offspring insulin resistance was programmed by IUGR with infantile overnutrition through higher methylation and lower expression of the peroxisome proliferator-activated receptor-gamma coactivator-1alpha (PGC-1α) gene (159). Increased methylation and decreased expression of glucose transporter 4 (Glut4) gene at the MYOD-binding site in gastrocnemius muscle of rat was reported to be induced by phthalate exposure *in utero*, associated with impaired insulin signaling (160). In a neonatal overfeeding mouse model, epigenetic programming of histone modifications at Monoacylglycerol O-acyltransferase 1 (Mogat1) locus was reported to link neonatal overnutrition with long-term hepatic insulin resistance and steatosis through increasing intracellular diacylglycerol content (161). In mice skeletal muscle, insulin sensitivity linked hypomethylation and increased expression of the Nr4a1 gene were reported to be programmed by the maternal high-fat diet and modulated by voluntary exercise in mice (162). Hepatic H3K14ac and H3K9me3 were reported to be significantly increased in WT and Glut4 (±) fetal and 5-week murine offspring with maternal high-fat-diet exposure *in utero* (155). Pathway analysis of ChIP-on-chip data revealed differential H3K14ac and H3K9me3 enrichment along pathways that regulate lipid metabolism, specifically in the promoter regions of Pparg, Ppara, Rxra, and Rora.

It was reported that elevated S-adenosylhomocysteine in adipocytes *in vitro* induced changes of DNA methylation, trimethylated histone H3-Lys27, and expression in genes involving glucose disposal and lipolysis pathway (163). In adipocytes differentiated from mesenchymal stem cells (MSCs) in Wharton's jelly of umbilical cord tissue of SGA neonates, it was reported that the increased acyl-coenzyme A synthetase 1 (ACSL1) (key roles in lipid metabolism) was highly associated with histone acetylation and could be a programmable mediator of insulin sensitivity and cellular lipid content (164).

Hepatic decreased miR-122 and increased miR-370 in mice offspring were reported to be induced by a maternal high-fat diet (165). Expression of insulin signaling associated hepatic miR-29b, miR-103, and miR-107 were reported to be increased in lambs with maternal obesity, and be ablated by maternal weight loss in the periconceptional period (166). Downregulation of IRS-1 in adipose tissue of offspring of obese mice was reported to be programmed cell-autonomously through increased miR-126 (167).

### Epigenetic Programming of Pancreas Islet Beta Cell's Development

More and more studies revealed that DNA methylation, histone acetylation (168), non-coding RNA and other epigenetic modifications were involved in the development and differentiation of pancreatic beta cell (63, 169–171). When exposed to the adverse intrauterine environment, fetal development programming of pancreatic islet beta cells happens, leading decreased number and/or function of pancreatic beta cells.

Histone modifications and associated chromatin patterns were reported in regulatory elements of silent genes that are activated upon pancreas development fate choices (172). In this process, the histone acetyltransferase P300 and the histone methyltransferase Ezh2 had modulatory roles in the fate choice. This study revealed a functional “prepattern” of chromatin states within multipotent progenitors and potential targets to modulate cell fate induction in pancreas development. Another research reported Dynamics of genomic H3K27me3 domains during pancreatic endocrine specification and Ezh2 as a critical determinant of endocrine progenitor number (173).

During healthy neonatal life after birth in mice, DNA methylation, especially DNMT3A, was reported to play a role to direct the acquisition of pancreatic beta cell function of glucose-stimulated insulin secretion (GSIS) (174). In this study, the metabolic switch associated encoding hexokinase 1 (HK1) gene and lactate dehydrogenase A (LDHA) gene were reported to be bound and methylated by DNMT3A. Knockdown of these two genes restored the GSIS response in islets from animals with beta cell-specific Dnmt3a deletion. DNA methylation of Pdx-1(175, 176) was also reported playing an important role in the development and function of pancreatic beta cells (170, 177).

Increased miRNA-199a-3p and miRNA-342 were reported to cause the decrease of the mTOR signaling pathway, and thereby reduce the number of beta cells and insulin secretion (63, 178). Long noncoding RNA H19 was reported to play an important role in postnatal β-cell mass expansion in rats and contribute to the mechanisms compensating for insulin resistance in obesity (179). Low-moderate exercise in obese fathers was reported to induce partial restoration of pancreatic islet cell morphology and the expression of pancreatic microRNAs (let7d-5p, 194-5p) in male offspring (180).

### Transgenerational Epigenetic Programming

Transgenerational epigenetic programming was considered a critical underlying mechanism of transgenerational inheritance caused by environmental interventions (2, 67, 181–183).

The concept of transgenerational epigenetic programming is proposed as epigenetic programming in germ cells. Twice genome-wide demethylation and further remethylation happen, respectively, during the development of germ cell and preimplantation embryo (6). The time of the development of germ cells and preimplantation embryos is considered to be important “windows” which is sensitive to epigenetic DNA methylation modification (184). Also, non-encoding RNA is involved in the transgenerational epigenetic programming (185, 185, 186). Such a concept means that the phenotype changes of F2 generation offspring after birth are caused by epigenetic programming in germ cells of F1 generation fetuses when exposed to the adverse intrauterine environment in the F0 generation.

Transgenerational epigenetic programming has been reported to be involved in a variety of phenotypes, including diabetes (4, 66), insulin resistance (3, 187), hypertension (188), reproduction (189), brain development (190, 191), and depression (185).

More and more researches supported the concept of transgenerational epigenetic programming of metabolic syndrome (4, 66, 183, 192). It was disclosed that transgenerational epigenetic programming could mediate the fetal programming of insulin resistance of insulin resistance, which was induced by high-fat diet, obesity or other interventions in F0 offspring (3, 68, 125). Transgenerational epigenetic programming was reported in adipose tissue development (5, 192). Also, transgenerational inheritance of obesity consistent with a leptin resistant was reported to be induced by transgenerational epigenetic programming of DNA methylome in adipose tissue (193–195).

Significant differential methylation changes in the promoter region of H19 were reported in a multigenerational model of intrauterine growth restriction (IUGR) (120).

## Conclusion

Fetal epigenetic programming is a concept that the intrauterine environment induced fetal epigenetic modification and associated gene expression activity is relatively stably transmitted after birth until adults, and thereby decide the physiological phenotype in adult from fetal development. Strong evidence in this review indicates that fetal epigenetic programming could be a critical underlying mechanism of fetal metabolic programming, which induces the circle of metabolic syndrome across generations.

Epigenetic modification involved in fetal metabolic programming of metabolic syndrome has been discussed above and demonstrated in Table 1. Obesity and insulin resistance were the key factors resulting metabolic syndrome (Figure 1). In brief, the detailed mechanism includes genes involved in adipose tissue development (IGF2/H19, IGF1R, Zfp423, FGF21, FAS), hyperphagia and energy balance regulation (leptin, POMC, GR, WNT2, TRβ), lipid metabolism (leptin, adiponectin, lipoprotein lipase, Cpt1a, PEPCK, Cpt1a), insulin associated signaling (ER, HIF3A, FAR2, PGC-1α, Glut4, Nr4a1), adipocytokine and proinflammatory factors (adiponectin, C3, LY86), pancreas islet beta cell's development (HK1, LDHA, Pdx-1), and DNA or histone-modifying enzymes (Dnmt3a, HDAC3, SIRT1,Phf2, G9a, P300, Ezh2). These gene expression changes through fetal epigenetic modification, which was induced by the adverse intrauterine environment, and thereby contributes metabolic syndrome in adult.

**Table 1:** Epigenetic programming in fetal metabolic programming.

**Fetal programming**		**Methylation**	**Histone modification**	**Non-coding RNA**
Obesity	Human study	Genome-wide epigenome^1^^,^^2^^,^^3^^,^^4^; abnormal birth weight, epigenome^1^^,^^2^^,^^3^^,^, IGF2/H19^Δ^^,^^1^^,^^2^, IGF1R^2^, energy homeostasis genes^1^, WNT2^3^, Glucocorticord receptor(GR)^3^, fat mass- and obesity- associated genes^3^, Cardiometabolic Risk genes^3^, POMC^1^; body composition, lipoprotein lipase^3^; adipocytokine, leptin^1^, adiponectin^2^		ex-miR-122^10^
	Animal model	Genome-wide epigenome^4^^,^^5^^,^^6^; abnormal birth weight, IGF2/H19^Δ^^,^^*^^,^^5^^,^^7^; adipocytokine, adiponectin^*^^,^^2^^,^^4^; hyperphagia and energy balance regulation, leptin^*^^,^^2^^,^^4^, POMC^8^, glucocorticoid receptor (GR)^8^; adipocyte differentiation and maturity, Dnmt3a^#^^,^^4^, Zfp423^4^; hepatic fat/steatosis, Cpt1a^5^	Genome-wide epigenome^5^; adipocytokine, adiponectin^*^^,^^2^^,^^4^; histone acetylation pathway genes^#^^,^^3^^,^^5^, SIRT1^#^^,^^5^; hyperphagia and energy balance regulation, leptin^*^^,^^2^^,^^4^, thyroid hormone receptor-β (TRβ)^8^; adipocyte differentiation and maturity, Zfp423^4^, G9a^#^^,^^4^, FGF21^4^, fatty acid synthase (FAS)^5^; hepatic fat/steatosis, PEPCK^5^, Cpt1a^5^, Phf2^#^^,^^5^, transcription factor Carbohydrate Responsive Element (ChRE)^5^,	miRNA-let-7g^6^
	*In vitro*	Genome-wide epigenome^9^; adipocyte differentiation and maturity, Dnmt3a^#^^,^^4^, obesity-related genes^4^		
Insulin resistance	Human study	Genome-wide epigenome^2^^,^^4^^,^^5^; adipocytokine and proinflammatory factors, adiponectin^3^, C3^3^; immune genes, LY86^2^; insulin associated signaling, ER^2^, HIF3A^2^^,^^4^, FAR2^2^; energy balance regulation, POMC^1^	HDAC3^#^^,^^2^,	ex-miR-122^10^, miR-15a^6^, miR-15b^6^,
	Animal model	Genome-wide epigenome^5^; Dnmt3a^#^^,^^4^, FGF21^4^; adipocytokine, adiponectin^4^, DNMT1^#^^,^^4^; insulin associated signaling, PGC-1α^5^^,^^6^, Glut4^6^, Nr4a1^6^	Genome-wide epigenome^5^; adipocytokine, adiponectin^4^^,^^3^; insulin associated signaling, Mogat1^5^; lipid metabolism, Pparg, Ppara, Rxra, and Rora^5^	miR-122^5^, miR-370^5^, miR-29b^5^, miR-103^5^, miR-107^5^, miR-126^4^,
	*In vitro*	Dnmt3a^#^^,^^4^; glucose disposal and lipolysis pathway genes^4^	Genome-wide epigenome^4^; lipid metabolism, ACSL1^4^	
pancreas islet beta cell's development	Animal model	glucose-stimulated insulin secretion (GSIS), DNMT3A^#^^,^^7^, HK1^7^, LDHA^7^; pancreas development, Pdx-1^7^;	Genome-wide epigenome^7^; pancreas development, P300^#^^,^^7^, Ezh2^#^^,^^7^, regulatory elements of silent genes^7^	miRNA-199a-3p^7^, miRNA-342^7^, miRNA-let7d-5p^7^, miRNA-194-5p^7^, lncRNA H19^7^,

Δ *Gene imprinting*,

* *transgenerational epigenetic programming*,

# *DNA or histone-modifying enzyme*.

1 *Cord blood*,

2 *peripheral blood leukocytes*,

3 *placenta*,

4 *adipose tissue*,

5 *liver*,

6 *muscle*,

7 *pancreatic islet*,

8 *hypothalamus*,

9 *oocytes*,

10 *circulating extracellular RNAs*.

There are still some unknowns and challenges in fetal epigenetic programming. Firstly, the molecular mechanism and regulation of epigenetic programming have not been fully disclosed. More key points and details are still required to be discovered. Secondly, results of related experiments sometimes could be disturbed by extra environmental disturbances not designed in experiments. Thirdly, epigenetic programming easily happens in susceptible “windows.” However, the threshold value of the adverse intrauterine environment is still not very clear in different types of diseases. Fourthly the size of epigenetic modifications is generally small in the whole epigenome. It may be caused by notable epigenetic modifications in fewer selectively targeted genes along with tiny epigenetic modifications in most non-targeted genes. The mechanism underlying the selective modifications is still needed to research. Beside of the selective epigenetic modifications around targeted gene region, the effects of more epigenetic modifications in other regions have not been well disclosed with their biological significance. Lastly, more importantly, there still are some limitations in the causality between epigenetic modifications and metabolic syndrome. There are a great deal of evidences of causality between epigenetic modifications and metabolic syndrome contributed by prospective clinical studies, animal and cell experiments in which the phenotypes of metabolic syndrome changed through the inhibition of DNA or histone modifying enzymes, and Chromatin immunoprecipitation assays identifying sequence-specific DNA binding protein which help to selectively modificate DNA or histone in a single gene's particular sequence. However, no molecular techniques have been invented to give direct logical evidence proving that metabolic syndrome could be induced or blocked by adding or erasing the epigenetic selectively modification in a particular sequence of a single gene. Here lies the most valuable breakthrough in epigenetic studies in future.

In spite of such unknowns, limitations, and challenges, the study of epigenetic programming in fetal metabolic programming has been developing rapidly, accompanied with more and more supporting evidence. It will not be surprising if future breakthroughs come soon in this field.

## Author Contributions

ZZ and XL conceived and initiated this study. ZZ contributed to writing of the manuscript. FC participated in conception and writing of this manuscript.

### Conflict of Interest

The authors declare that the research was conducted in the absence of any commercial or financial relationships that could be construed as a potential conflict of interest.
